# Transitioning health workers from PEPFAR contracts to the Uganda government payroll

**DOI:** 10.1093/heapol/czab077

**Published:** 2021-07-08

**Authors:** Henry Zakumumpa, Joseph Rujumba, Woldekidan Amde, Respicius Shumbusho Damian, Everd Maniple, Freddie Ssengooba

**Affiliations:** School of Public Health, University of the Western Cape, Private Bag x17, Bellville, 7535 Republic of South Africa; Makerere University, School of Medicine, P O Box 7062, Kampala, Uganda; School of Public Health, University of the Western Cape, Private Bag x17, Bellville, 7535 Republic of South Africa; Faculty of Social Sciences, University of Dar es Salaam, P O Box 35091, Dar Es Salaam, Tanzania; School of Medicine, Kabale University, P O Box 317, Kabale, Uganda; Makerere University, School of Public Health, P O Box 7072, Kampala, Uganda

**Keywords:** Human Resources for Health, health systems, decentralization, HIV, donor transition, implementation research, overseas development assistance

## Abstract

Although increasing public spending on health worker (HW) recruitments could reduce workforce shortages in sub-Saharan Africa, effective strategies for achieving this are still unclear. We aimed to understand the process of transitioning HWs from President’s Emergency Plan for AIDS Relief (PEPFAR) to Government of Uganda (GoU) payrolls and to explore the facilitators and barriers encountered in increasing domestic financial responsibility for absorbing this expanded workforce. We conducted a multiple case study of 10 (out of 87) districts in Uganda which received PEPFAR support between 2013 and 2015 to expand their health workforce. We purposively selected eight districts with the highest absorption rates (‘high absorbers’) and two with the lowest absorption rates (‘low absorbers’). A total of 66 interviews were conducted with high-level officials in three Ministries of Finance, Health and Public Service (*n* = 14), representatives of PEPFAR-implementing organizations (*n* = 16), district health teams (*n* = 15) and facility managers (*n* = 22). Twelve focus groups were conducted with 87 HWs absorbed on GoU payrolls. We utilized the Consolidated Framework for Implementation Research to guide thematic analysis. At the sub-national level, facilitators of transition in ‘high absorber’ districts were identified as the presence of transition ‘champions’, prioritizing HWs in district wage bill commitments, host facilities providing ‘bridge financing’ to transition workforce during salary delays and receiving donor technical support in district wage bill analysis—attributes that were absent in ‘low absorber’ districts. At the national level, multi-sectoral engagements (incorporating the influential Ministry of Finance), developing a joint transition road map, aligning with GoU salary scales and recruitment processes emerged as facilitators of the transition process. Our case studies offer implementation research lessons on effective donor transition and insights into pragmatic strategies for increasing public spending on expanding the health workforce in a low-income setting.

## Key messages

- Providing technical support in conducting district wage bill analyses can reveal unutilized funds for workforce recruitments in Low- and middle-income countries (LMICs).
- Aligning with donor–recipient government salary scales and operating within established recruitment procedures enhances chances of enrolment of donor-contracted health workers onto the public sector payroll.
- Targeted donor aid can help unblock systemic barriers to expanding the health workforce in decentralized settings in LMICs.
- Multi-sectoral engagements incorporating the Ministry of Finance are critical in realizing effective donor transitions.

## Introduction

Health workforce shortages constitute a fundamental barrier to the attainment of health-related sustainable development goals in sub-Saharan Africa (SSA) (Freer, 2017). It is estimated that health worker (HW) vacancies are as high as 59–70% in several countries in SSA (Vermund *et al.*, 2012; Zakumumpa *et al.*, 2016; Jaskiewicz *et al.*, 2016).

In countries with decentralized health systems in SSA, health workforce recruitments have been devolved from the central government to sub-national administrative units commonly referred to as districts in Ghana, Nigeria, Tanzania and Uganda (Bossert and Beauvais, 2002; Munga *et al.*, 2009; Sakyi, 2013; Abimbola *et al.*, 2019; Sumah and Baatiema, 2019).

The recruitment of HWs in district health systems in SSA is however beset by a myriad of institutional constraints. These include delays in recruitment owing to cumbersome and lengthy administrative procedures, the ubiquitous ‘vacancies but no wage bill’ constraint and a shortage of payroll analysis expertise (Munga *et al.*, 2009; Sakyi, 2013; Frumence *et al.*, 2013; Mbemba *et al.*, 2016; Sumah and Baatiema, 2019).

In Uganda, decentralization was a part of governance reforms that date as far back as 1992 (Awortwi, 2010; Tashobya *et al.*, 2016). Health sector decentralization was formally provided for under the 1995 national constitution and further operationalized in the Local Government Act of 1997 (Tashobya *et al.*, 2016). Uganda’s district health system comprises of a district hospital and lower-level primary care health facilities (Alonso-Garbayo *et al.*, 2017). In 2014, management of the public sector payroll processing system was further decentralized to districts (Lwanga *et al.*, 2018). District recruitment bodies known as ‘district service commissions’ conduct interviews and selections of HWs. However, devolved recruitment, in practice, is a shared responsibility between the districts (which declare vacancies and make recruitment decisions) and relevant central government line ministries such as Finance and Public Service which commit funds to the public sector wage bill. There exists a heavy dependence by districts on central government grants for both capital development and basic operational funds (Alonso-Garbayo *et al.*, 2017).

HW shortages are pervasive at all levels of the health system in Uganda (Zakumumpa *et al.*, 2017). In 2012, the Uganda government implemented an aggressive health workforce recruitment programme known as ‘the surge’ in which 7112 HWs were recruited to plug severe staffing gaps at the primary care level (Jaskiewicz *et al.*, 2016). Specifically, HWs were recruited at the level of Health Centre IVs (sub-district) and Health Centre IIIs (sub-county). However, severe staffing gaps remained at the level of district hospitals that have relatively high Human Immunodeficiency Virus (HIV) client loads (Zakumumpa *et al.*, 2016). In response to these staffing gaps, and in order to accelerate progress towards HIV epidemic control in Uganda, President’s Emergency Plan for AIDS Relief (PEPFAR) developed a 3-year ‘Human Resources for Health Support Program’ in 2013 that was implemented in 87 focus districts (USAID, 2019). According to the implementation plan, the HWs would be initially recruited on contract by PEPFAR for a period of 2 years but would subsequently be absorbed into the mainstream public service as soon as fiscal space allowed. A total of 3154 HWs were recruited by PEPFAR in Uganda between 2012 and 2015. Of these, 694 were enrolled onto the Government of Uganda (GoU) payroll between 2013 and 2017 (USAID, 2019). An additional 1965 HWs were expected to be transitioned to the GoU in a phased manner between 2017 and 2020.

There has been increasing international assistance in addressing the human resources for health crisis in SSA in the quest to sustain public health gains during periods of donor support and to sustain these outcomes during donor transition (Micah *et al.*, 2018). This has motivated analyses by external donors and recipient governments around increasing reliance on domestic financing (Amaya *et al.*, 2014; Bennett *et al.*, 2015; Burrows *et al.*, 2016; Vogus and Graff, 2015; Resch and Hecht, 2018; Gotsadze *et al.*, 2019). Fiscal space has been defined as ‘the capacity of government to provide additional budgetary resources for a desired purpose without any prejudice to the sustainability of its financial position’ (Heller, 2006). As donors like PEPFAR reduce their financial support to workforce costs, little is known about which factors hinder or facilitate increases on public spending on HW recruitments within the government. These data are critical to understanding the dynamics involved and strategies needed for increasing domestic financial responsibility and local ownership by recipient countries (Vermund *et al.*, 2012; Palen *et al.*, 2012; Amaya *et al.*, 2014; Bennett *et al.*, 2015; Vogus and Graff, 2015; Burrows *et al.*, 2016; Resch and Hecht, 2018).

Although there is an accumulating evidence base on the notion of decision space in district health systems, in general (Bossert and Mitchell, 2011; Liwanag and Wyss, 2018; Henriksson *et al.*, 2019; Bulthuis *et al.*, 2021), and around Human Resources for Health in particular (Alonso-Garbayo *et al.*, 2017; Sumah and Baatiema, 2019), there is little empirical attention to the prospect of increasing public spending on expanding the health workforce in decentralized settings in low-income countries. We aimed to understand the process of transitioning HWs from PEPFAR contracts to the Uganda government payroll and to explore the facilitators and barriers encountered in increasing domestic financial responsibility for this transition.

## Materials and methods

### Research design

We utilized a qualitative case-study research design. Case studies are recommended for an in-depth understanding of complex phenomena within organizations (Yin, 2003; Gilson, 2012). We conducted a multiple case study of 10 districts in Uganda categorized into two: (1) eight districts with the highest rates of absorption of HWs recruited with PEPFAR support dubbed ‘high absorbers’ cases and (2) two districts with the lowest absorption rates or the ‘low absorbers’ cases. We then conducted a comparative analysis across the two categories of cases with regard to facilitators and barriers to HW transition.

### Case-study selection

The 10 case-study districts were purposively selected from 87 districts in Uganda, which received PEPFAR support in recruiting HWs between 2013 and 2017. Study districts were purposively selected based on secondary analysis of databases in the Human Resources Information System (HRIS) and a locally based international PEPFAR-implementing organization’s databases of HWs recruited between 2015 and 2017. From these databases, we selected districts with the highest number of HWs transitioned from PEPFAR to GoU payrolls. Table 1 shows we selected districts with the highest number of HWs transitioned from each of eight geographic sub-regions as defined by the Uganda Bureau of Statistics (Iganga, Sheema, Apac, Kasese, Napak, Nwoya, Tororo and Kampala) and based on HIV burden (which was the key focus of PEPFAR support). We aimed to achieve diversity in our sample of districts by setting (rural/urban). The detailed inclusion criteria are described Table 1. Due to logistical limitations, we selected only two districts with the lowest number of HWs absorbed onto their payroll (Nakaseke and Bushenyi). Each of the two districts had absorbed only ‘one’ HW since 2013 when PEPFAR’s health workforce transition programme commenced in Uganda.

**Table 1.** Characteristics of case-study districts

Geographic sub-region of Uganda	District	HIV prevalence (%)	Inclusion criteria
South Western	Sheema	7.7	High number of transitioned HWs and high HIV prevalence
East Central	Iganga	4.4	High number of transitioned HWs, mixed rural–urban
Mid-East	Tororo	4.8	High number of transitioned HWs and cross-border dynamics, mainly urban
Mid-West	Kasese	5.5	High number of transitioned HWs and cross-border dynamics, mainly urban
Central 2	Mubende	7.4	High number of transitioned HWs and high HIV prevalence
Mid-North	Nwoya and Apac	7.0	High number of transitioned HWs and high HIV prevalence, largely rural
North East	Napac	3.4	High number of transitioned HWs and hard to reach, rural
Kampala	Kampala	6.6	Capital city and houses many agencies involved in transition planning

### Conceptual framework

We utilized an implementation research lens (Proctor *et al.*, 2011) to understand the process of transitioning HWs from PEPFAR to the Uganda government payroll and in order to explore the facilitators and barriers involved in this process. More specifically, we adopted the Consolidated Framework for Implementation Research (CFIR) as the analytical framework underpinning this study (Damschroder *et al.*, 2009). The CFIR is a ‘meta-theoretical’ framework that was informed by earlier implementation research frameworks. The CFIR is derived from a robust systematic review of factors influencing the implementation of ‘interventions’ (Means *et al.*, 2020). The CFIR framework provides a multi-level analysis lens that entails 39 constructs categorized under 5 ‘domains’ (‘intervention characteristics’, ‘outer setting’, ‘inner setting’, ‘characteristics of individuals’ and ‘process of implementation’). ‘Intervention characteristics’ refer to the attributes of the intervention itself such as perceptions of its effectiveness by stakeholders, its design quality, its adaptability and its cost-effectiveness. ‘Outer setting’ implies factors originating from the external environment such as those emerging from outside of the host implementing organization such as external policies and legal frameworks. ‘Inner setting’ refers to factors influencing the uptake of an intervention that derive from the internal organizational context such as culture, climate and readiness for implementation. ‘Characteristics of individuals’ refer to the personal attributes of the individuals involved in implementing the intervention such as their self-efficacy, knowledge levels, competence and value systems. ‘Process of implementation’ refers to the various stages involved in rolling out the intervention and its influence on implementation success such as the quality of planning and level of stakeholder engagement. The CFIR guided this study in three ways. It informed the diverse range of study participants selected for this study, especially those involved in the transition of the contract workforce onto the public sector payroll. It helped in constructing our qualitative interview guides during data collection and provided an overarching deductive thematic framework for our synthesis and interpretation of study findings and in their presentation (Means *et al.*, 2020).

### Data collection

In keeping with the CFIR framework’s multi-level analysis lens, we selected study participants involved in the transition process at the ‘policy and planning’, ‘programmatic’ and ‘implementation’ levels: (1) national-level policy and planning actors, e.g. sector ministry officials (Ministry of Health, Finance and Public Service) and PEPFAR as well as its ‘implementing partner (IP)’ playing the overall national coordination function of overseeing the transition process; (2) sub-national operational-level actors [e.g. district health officers (DHOs), district personnel officers and PEPFAR-implementing organizations at the sub-national operational level]; (3) facility-level actors (hospital administrators and the principal nursing officers (head nurse) and transitioned HWs across diverse cadres. The category of participants is shown in Table 2.

**Table 2.** Category of participants

Respondent type	Round 1	Round 2	Total
High-level sector ministry technocrats	14	0	14
District health team leaders	12	3	15
Facility in-charges/managers	18	4	22
Representatives of regional-based PEPFAR-implementing partners (IPs)	11		13
U.S. embassy programme officers (USAID and CDC)	3	0	3
Focus group discussions	6	2	15
Transitioned HWs	75	12	87

USAID, United States Agency for International Development; CDC, Centers for Disease Control.

We conducted 15 face-to-face key informant interviews (KIIs) with national-level actors who had ‘insider’ insights into the HW transition process right from inception [e.g. during the signing of memorandums of understanding (MoUs)] such as the overall coordinating PEPFAR-implementing organization, programme officers in the United States embassy in Uganda and high-level technocrats in line Ministries of Health, Finance and Public Service who were directly involved in the inception meetings and in the consensus building between PEPFAR and the Uganda government around absorption of HWs after their 2-year contract period.

We then conducted 24 in-depth interviews with district-level actors in 10 case-study districts who were directly involved in the implementation of health workforce transition at the sub-national level. These included DHOs and district human resources officers. The interview guide used in our interviews was constructed around the five CFIR-derived domains (‘process of implementation’, ‘intervention characteristics’, ‘outer setting’, ‘inner setting’ and ‘characteristics of individuals’). This overarching framework helped in eliciting the facilitators and barriers to HW transition. Data were collected over two rounds. For the ‘high absorber’ cases, data were collected between June and September 2018 (round 1) and January to March 2020 (round 2) among the ‘low absorber’ case districts.

Twelve focus group discussions (FGDs) were conducted with 87 HWs who were transitioned onto the public payroll in the case-study districts to better understand transition enablers and barriers from their perspective. Ten focus groups were conducted in ‘high absorber’ districts, while two FGDs were conducted in ‘low absorber’ districts. The interviews were conducted in English by HZ and JR, with the assistance of four research assistants who operated the recorder and took notes.

To augment respondent data, we conducted a desk review of relevant documents such as ‘PEPFAR’s Human Resources for Health (HRH) Support for Recruitment—Implementation plan of April 2013’. We reviewed written memos from two central government line Ministries of Public Service and Health addressed to district local government leaders, urging them to absorb the health workforce recruited with PEPFAR support.

### Data analysis

Qualitative data were analysed in line with the procedures recommended by Miles and Huberman (1994). Interviews were recorded in English, transcribed verbatim into text transcripts by four research assistants. Data were analysed, in an iterative process, involving four major steps: (1) ‘data familiarization’ (HZ, JR read the interview transcripts multiple times); (2) ‘developing a coding framework’: we adopted the five CFIR-derived domains (‘intervention characteristics’, ‘outer setting’, ‘inner setting’, ‘characteristics of individuals’ and ‘process of implementation’) as an overarching deductive thematic framework as well as inductively, from the data (Fereday and Muir-Cochrane, 2006); (3) ‘data abstraction’ of the coded data into thematic categories while engaging in a constant comparative analysis across the two categories of cases of ‘high absorber’ and ‘lower absorber’ districts (Glaser and Strauss, 1999) and (4) ‘overall interpretation and synthesis’: the final analyses were reached by consensus in a process involving at least four of the authors.

In addition, we adopted the recommended procedures for ensuring rigour in case-study analysis (Gilson *et al.*, 2011).

## Results

The identified facilitators and barriers to HW transition emerging from this study are presented based on the five CFIR-derived domains.

### Process of implementation

Table 3 shows the milestones in the process of implementation of the HW transition process. Implementation happened at three major levels: (1) national-level ‘policy planning and coordination’, (2) sub-national level ‘programmatic supervision’ and (3) facility-level ‘implementation’.

**Table 3.** Milestones in the HW transition implementation process

Level and stakeholders	Key actions
National	MoU between PEPFAR and GoU Harmonization of salaries Inter-sector transition meetings around a road map Developing a transition road map
District	Joint Planning by regionally-based IPs and district actors Determining district HRH needs Wage bill analysis HW recruitment and deployment
Health facility level	HW orientation HW performance management during the contract phase

#### National-level stakeholder engagement and transition planning

At the national level, the process involved consensus-building meetings between PEPFAR and GoU high-level actors around absorption of the recruited workforce after phasing out of support. This culminated in a formal MoU between the two parties. The MoU stipulated that PEPFAR would provide funds for the recruitment process and salary support for the initial 2 years, and GoU would subsequently enrol the recruited HWs on the public sector payroll as soon as fiscal space allowed. Inter-sector meetings were convened incorporating the relevant line Ministries of Health, Finance and Public Service. PEPFAR was represented by its overall national coordinating agency—an international non-governmental organization, which consulted with relevant programme officers at the United States embassy in Uganda. In 2013, a HW transition implementation plan and a road map were jointly agreed through a consultative process involving the two parties. Salary harmonization was a key point whereby PEPFAR would pay the recruited workforce (during their 2-year contract phase) salaries that were comparable to GoU salary scales. With the exception of payment of an housing allowance to the contract workforce, PEPFAR’s pay structure was well aligned with the GoU. The PEPFAR national coordinating agency continually monitored the transition process and regularly shared insights and progress reports with the Ministry of Health’s Human Resources for Health Technical Working Group.

At the district level, regionally based PEPFAR IP organizations in the 87 focus districts in Uganda held transition planning meetings that engaged district-level actors such as district health teams, chief administrative officers (CAOs) and district human resource officers as well as district service commissions (DSCs), which make personnel selection decisions. During such meetings, a transition road map at the district level and the roles of the varied stakeholders were agreed upon. District health teams in conjunction with district personnel offices determined the HW cadres to be recruited based on the needs of individual districts. Table 4 shows that the bulk of HWs recruited across case-study districts were midwives, nurses and clinical officers. These vacancies were advertised in national newspapers and through district and facility notice boards. The processes of initial formal recruitment were led by the districts with the financial support of PEPFAR provided through its regionally based IPs. Across all districts, contract staff were vetted by the DSCs to ensure that they met the Uganda public service standards for recruitment. The IPs managed contracts and payrolls during the 2-year contract phase for the transition workforce. In most of the 87 focus districts, an independent PEPFAR contractor was mentioned as the personnel contracts and payroll management agency. The district health teams together with IPs monitored the performance of contract HWs through instruments such as time sheets that were a basis for approving salaries.

**Table 4.** Cadres of HWs transitioned from PEPFAR to Go U

Health worker cadre	No. of transitioned HWs	% by HW cadre *n* = 694
Enroled nurse	275	39.6
Enroled midwife	204	29.4
Medical laboratory technician	54	7.8
Medical clinical officer	50	7.2
Biostatistician	35	5.0
Medical officer	30	4.3
Nursing officer nurse	14	2.0
Medical laboratory technologist	13	1.9
Enroled comprehensive nurse	8	1.2
Nursing officer midwife	6	0.9
Dispenser	3	0.4
Pharmacist	1	0.1
Medical records assistant	1	0.1
Laboratory assistant	0	0.0
Anesthetic officer	0	0.0
Total	694	99.9

At the facility level, contract staff were oriented in public service processes by their immediate supervisors. The district health teams and facility service managers were instrumental in providing supervision and appraisal of contract staff. This formed the basis of the selection of HWs on contract who were to be absorbed onto the public payroll.

### Characteristics of the intervention

#### District wage bill budget analysis support

Although PEPFAR support triggered the creation of 694 ‘new’ wage bill slots for additional HWs in intervention districts, our findings suggest that a small part of these slots was derived from the unutilized wage bill in district budgets.

Technical support for district wage bill analyses was extended by the coordinating PEPFAR-implementing organization to districts. This was reported as a facilitator of the HW transition process in ‘high absorber’ districts. Although there was a widely held perception, among actors within the district administrations that their budgets could not accommodate any new personnel recruitments, technical support in scrutinizing district wage bills revealed unutilized funds that were subsequently committed to absorbing the contract workforce.

*PEPFAR helped us analyze the wage bill budget. There was some confusion with the Ministry of Public service and Ministry of Health and here at the district. We were in the dark. So, PEPFAR came and analyzed and found that we had a balance (funds for salaries) which we were not using* (KII, district official, Iganga).

Conversely, in ‘low absorber’ districts, participants reported that they did not receive technical support in wage bill analyses. Hence, donor support in wage bill analyses emerged as a distinguishing feature between the two categories of ‘high absorber’ and ‘low absorber’ districts.

**Figure 1.**
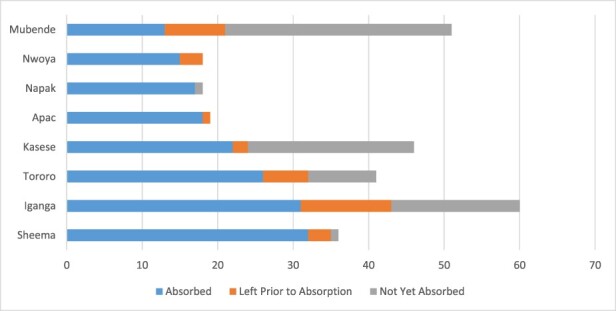
Number of HWs absorbed in ‘high absorber’ districts

However, Figure 1, which is generated from the secondary analysis of the HRIS database, shows that even across the ‘high absorber’ districts there were still a significant number of contract staff who were not enrolled onto the public sector payroll. Wage bill ceilings limited the ability of districts to absorb a higher number of contract staff. We observed that ‘low absorber’ districts had a higher number of their contract staff seconded to private not-for-profits (PNFPs) such as mission hospitals that had an even weaker absorption capacity (at 30%) compared to district local governments (at 55%).

*The absorption has been very slow in PNFPs because these did not have money to absorb them. Most PNFPs were comfortable offering services with low cadre staff and do not have a budget to hire high cadre staff. But PEPFAR hired these staff for the HIV response but health facilities do not have income to maintain them* (KII, national-level official).

**Figure 2.**
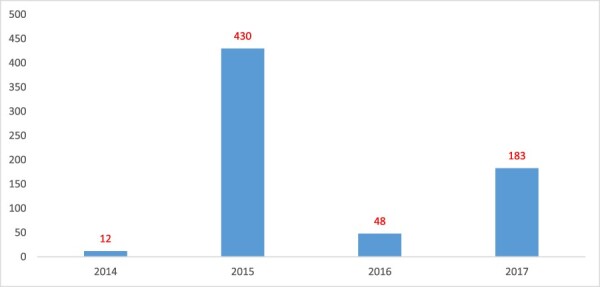
Number of HWs transitioned between 2014 and 2017

Secondary analyses of HRIS and PEPFAR databases revealed that over 500 of the recruited workforce were not absorbed in GoU service after transition. Figure 2, which is derived from these secondary analyses, shows the number of HWs absorbed between 2012 and 2017. Across case-study districts, a number of HWs left government service before they were formally absorbed. In the focus group, HWs shed light on why this was the case. The reasons include prolonged delays in accessing the public payroll after their 2-year PEPFAR contracts had run out, a lack of private accommodation (especially in rural Northern Uganda) and challenging work environments such as chronic stock-outs of supplies. Many of the unabsorbed HWs opted for alternative employment—mostly in the private sector.

#### Support in convening district personnel recruitment committees

PEPFAR support helped in unlocking long-standing organizational barriers to the expansion of the existing health workforce in ‘high absorber’ cases. A lack of basic operational funds for supporting the lengthy procedures required for hiring new staff was a constraint raised across all case-study districts. DSCs are standing committees that make personnel selection decisions and are meant to sit every 3 months. However, the DSCs were widely reported to be dysfunctional owing to a chronic inability to raise monetary allowances for paying the non-full-time DSCs that are comprised of retired senior public servants. Running district job adverts in national newspapers was said to be prohibitively expensive. PEPFAR provided the necessary funding to kick-start recruitment processes in form of paying for newspaper job adverts, providing monetary allowances to DSCs and sent observers to meetings where job interviews were conducted, which enhanced transparency and objectivity in the selection processes. United States embassy programme officers in Uganda reported that PEPFAR had committed $9 333 891 for the HW transition programme in 2012 alone and an additional $4 494 149 in 2015. District-level informants described the nature of PEPFAR support they received

*PEPFAR helped with providing the recruitment funds. It provided sitting allowances to enable District Service Commissions to convene as well as providing allowances to committee members during the interview of candidates. They facilitated most of the activities utilizing our own technical staff* (KII, district official, Sheema District).

#### Transparency in recruitment of the transitioned health workforce

A number of HWs reported that before the PEPFAR intervention, DSCs had a reputation of questionable objectivity in the selection of personnel due to a widely held perception that nepotism and bribery were common in district personnel recruitment decisions. Given this context, the selection of PEPFAR-supported HWs through transparent and merit-based processes lent special legitimacy to the transition workforce, which enhanced their absorption prospects into public service. District- and facility-level managers perceived PEPFAR-supported personnel as having been recruited through rigorous and objective procedures.

*I look at it as a good strategy for recruiting staff. This issue of our local politics of you are going to recruit this one’s daughter (nepotism), you are going to solicit bribes… those ones didn’t surface anywhere. It was a purified process that government didn’t have any reason whatsoever to object to their absorption. Someone recruited by an NGO interested in health you can’t doubt their qualifications, you can’t doubt their capabilities and then I think it also eliminated this issue of tribalism (ethnic biases) in recruitments* (KII, district official, Tororo).

The transitioned workforce was perceived as competent for absorption into government service. The 2-year contract phase funded by PEPFAR allowed facility-level managers to identify resilient and dependable HWs for absorption. In addition, this phase also provided HWs with an opportunity to be inducted and initiated into government systems and work environments.

*When they came the health workers on contract exhibited professionalism in their work. They were good people and immediately, they started working. The quality of service, was realized by the community. I think there is a visible change in the hospital since they came in* (KII, district official, Apac).

### Outer setting

#### Multi-sectoral engagements in transition process

At an institutional level, multi-stakeholder engagements involving actors at the national, sub-national and facility levels were identified as a major transition facilitator by participants in ‘high absorber’ districts. At the national level, PEPFAR was involved in multi-sectoral engagements of high-level actors with authority for approving HW recruitments in relevant central government sector ministries such as Finance, Public Service and Health. A transition road map and an MoU were agreed between PEPFAR and sector ministries in which PEPFAR undertook to provide salary support of the new workforce recruits for 2 years while the Uganda government would enrol these HWs onto the public sector payroll as soon as fiscal space permitted. This facilitated buy-in from influential actors in sector ministries. At the sub-national level, PEPFAR-implementing organizations in the various geographic sub-regions spearheaded engagement with sub-national actors such as district health officers and CAOs. MoUs were signed between regionally based PEPFAR-implementing organizations and the districts under their purview.

*We had several interactions. Ministry of Health invited us. As a district, we are supposed to implement Ministry of Health policies. The policy was such that PEPFAR would recruit those health workers on contract and with time, the districts, with help of Ministry of Health and Finance would avail a wage bill to absorb them* (KII, district official, Sheema).

Crucially, PEPFAR worked within established Uganda government recruitment processes and structures. Districts determined the cadres that would be hired based on their needs. DSCs made the ultimate hiring decisions. This lent legitimacy to the cohort of HWs recruited with PEPFAR support.

*All recruitment of contract staff was done by DSCs. So when it comes to absorption, such health workers are regularized because they were already recognized as legitimate staff hired through competent structures* (KII, national-level official).

### Inner setting

#### Prioritization of HWs in district recruitments

The prioritization of HWs in district personnel recruitments was a key distinguishing feature between ‘high absorber’ and ‘low absorber’ cases. In ‘high absorber’ districts such as Kasese and Sheema, participants were unequivocal in relaying the notion that their district administrations deliberately prioritized the health workforce in recruitments. In the ‘high absorber’ cases, whenever some fiscal space in the district wage bill emerged, slots for HWs were ‘ring-fenced’ as the overall priority. This took the shape of an informal recruitment policy.

*In fact we had to trade off some cadres, those ones who were not extremely needed or useful we had to keep them off in order to bring in the more useful staff like the midwives and clinical officers* (KII, district official, Apac).

*You may have the wage bill but how are you going to prioritize the cadres of peoples you are going to recruit? You may say all Health Centre IIs need a security guard. You may recruit like 20 porters. I know they are needed there but is it a priority?* (KII, district official, Sheema).

Although we found that ‘high absorber’ districts prioritized HWs in their wage bill, national-level informants reported that this was further reinforced by formal written memos. The memos originated from Ministries of Public Service and Health to the district political and technical leadership asking that they prioritize the absorption of PEPFAR-supported workforce in the available wage bill of the districts. These memos were written in March 2013 as a result of the protracted engagements by PEPFAR and high-level actors in sector ministries that were in line with the agreed transition road map.

### Characteristics of individuals

#### Presence of transition ‘champions’

The presence or absence of transition ‘champions’ differentiated between ‘high absorber’ and ‘low absorber’ districts. Whereas ‘high absorber’ districts reported the presence of internal transition ‘champions’, their absence in participant discourses in ‘low absorber’ districts was unmistakable. Transition ‘champions’ were individuals who went above and beyond the call of duty to promote the absorption of HWs onto the public payroll. These champions were reported at district and facility levels. The presence of champions at multiple levels created synergies in promoting HW absorption in ‘high absorber’ districts. The frequently cited champions include influential actors such as CAOs of host districts, DHOs and hospital administrators who actively pushed for the recruitment and absorption of HWs and enrolment on the government payroll.

*We had a smooth transition because the team in XXX (District) is very proactive. They don’t operate like they are in government. The CAO (Chief Administrative Officer) was an experienced man so he was quick to come in and push the recruitment process along. Much faster than is normally the case. The District Human Resource Officer was very active. They did their work in a timely way and actively pushed to have the HWs absorbed* (KII, district official, Iganga).

Champions tirelessly worked to expedite processes in the context of the typically lengthy administrative procedures in the Ugandan public sector. They acted as ‘persistence enhancers’ for HWs and even appropriated district finances to create ‘stop-gap’ monetary allowances for HWs before they were able to access the public payroll.

Actors at the facility level were frequently cited as transition champions. Facility in-charges were motivated by a need to avoid losing skilled HWs who had been posted to their health facilities. As such, they were instrumental in ensuring timely appraisal of contract HWs but also engaged in active follow-up with DSCs at the district administration headquarters for absorption of HWs. Facility in-charges in ‘high absorber’ districts actively engaged their transition workforce in activities such as surgical camps and community outreaches to enable them secure some field monetary allowances to sustain them as they awaited enrolment on the payroll.

*The salaries could delay for two to three months. We have PHC (primary health care) funds earmarked to this facility. We used some of this to buy them basics such as soap and sugar that could also help them to persist and endure* (KII, facility in-charge, Nwoya district).

## Discussion

We conducted a multiple case study of 10 districts in Uganda to better understand why they had variations in absorption rates of the health workforce transitioned from PEPFAR payroll support. We found distinguishing features between the two ‘low absorber’ districts and the eight ‘high absorber’ districts. We found that in the latter cases, conducting a wage bill analysis of district budgets to discover unutilized funds, the presence of transition ‘champions’ and prioritizing HWs in the available district wage bill differentiated them from the ‘low absorber’ districts where these attributes were absent. At an institutional level, multi-stakeholder and multi-sectoral engagements, agreeing on a joint transition road map and PEPFAR’s alignment with Uganda government salary scales and recruitment procedures enabled over 694 HWs to be added to the public sector payroll. However, district wage bill caps, prolonged delays in enrolment onto the public sector payrolls and a lack of private accommodation for transition HWs were common across districts.

### Implementation research and strategies for effective donor transitions

The PEPFAR HW transition case studies documented here offer implementation research lessons on effective donor transition for other global health initiatives and bilateral development partners. We observe that, in this particular study, PEPFAR’s transition model conforms with three (of the six) donor transition ‘good practices’ that were earlier proposed (Vogus and Graff, 2015). More specifically, we found that agreeing on a joint transition road map, communicating early about the transition intentions and aligning with Uganda government salary scales and recruitment procedures enhanced health workforce absorption. In our analysis of participant discourses, we noted that there were insufficient monitoring and evaluation measures in the HW transition road map. There is a sparse but emerging evidence base on recommended donor transition planning and management in the health sector (Palen *et al.*, 2012; Amaya *et al.*, 2014; Bennett *et al.*, 2015; Vogus and Graff, 2015; Burrows *et al.*, 2016).

Although previous studies have noted PEPFAR’s strong vertical orientation in its support for national HIV responses, often provided within parallel structures to those of donor–recipient governments (Windisch *et al.*, 2011; Luboga *et al.*, 2016; Ssengooba *et al.*, 2017). We document a unique case study that runs contrary to previous PEPFAR intensely vertical aid approaches. There is little doubt that PEPFAR’s multi-stakeholder engagement resulted in increased Uganda government budgetary allocation for expanding the health workforce in case-study districts. PEPFAR’s health-system strengthening (HSS) intervention triggered the absorption of 694 HWs onto the public sector payroll. A notable finding of this study was that PEPFAR’s multi-sectoral engagement of high-level actors in Uganda generated buy-in from the influential Ministry of Finance, which technically commits votes in the national budget. Although there are mounting calls for engagement of Ministries of Finance in expanding fiscal space for health (Whyle and Olivier, 2016), there is little research documenting ‘catalyst’ engagements that result in tangible outcomes, especially with regard to addressing the human resources for health crisis. Our study suggests that sustained engagements with the Ministry of Finance and other line ministries helped 694 HWs transition to public sector payrolls. However, Bennett *et al.* (2018) highlight the influence of external development partners in providing the financial impetus for initiating multi-sectoral collaborations, and note that local actors may not have this leverage: ‘In LMIC, a related factor concerns the role of external development partners. Multi-sectoral action that has strong external support likely has better access to financial resources, but may suffer from limited local ownership (and hence perhaps low motivation), and conceivably organizational blue prints that do not align with ways of doing business in country.’

A review article assessing HSS interventions by Adam *et al.* (2012) recommends the application of a ‘systems thinking’ lens in designing HSS interventions that engage and cut across the six ‘building blocks’ or sub-components of a health system. In reflecting on participants’ discourses in this study, we observe that PEPFAR’s Human Resources for Health support in Uganda and the subsequent donor transition effort engaged with at least four intersecting health system ‘building blocks’, namely, health financing, health information systems, leadership and governance and health workforce (Van Olmen *et al.*, 2012; Mutale *et al.*, 2013; Mounier-Jack *et al.*, 2014; Zakumumpa *et al.*, 2018).

In this study, we found that PNFP facilities had lower absorption rates of the workforce transitioned from PEPFAR support at (30% absorption) compared to district local governments with a 55% absorption rate. Our study adds to accumulating calls for government support to the private sector in bolstering human resources for health, including payroll support (Zakumumpa *et al.*, 2016) and the need for increased engagement by donors and governments of the private sector in accelerating progress towards universal health coverage (Montagu *et al.*, 2016; Ssennyonjo *et al.*, 2018; Wilhelm *et al.*, 2020).

The adopted five domains of the CFIR framework were helpful in providing a broad deductive framework for our overall synthesis and interpretation of study findings as well as in their presentation—we note that although the framework categorizes into five domains, some of our findings appeared to cut across more than one domain. For instance, we found that the prioritization of HWs in district wage bill commitments derived from ‘inner setting’ priority setting but was re-enforced by ‘outer setting’ factors such as written memos from line ministries. In this sense, our study suggests some dynamic interactions in facilitators of HW transition in Uganda. The notion of dynamic interactions in factors influencing the implementation and sustainability of health programme interventions has been observed in previous studies (Durlak and DuPre, 2008; Stirman *et al.*, 2012; Zakumumpa *et al.*, 2018)

### The challenges of workforce recruitments in decentralized settings

This study illuminates the dysfunction that underpins recruitment freezes in decentralized settings in Uganda. This ranges from insufficient basic operational funds for convening recruitment bodies, limited expertise in wage bill analysis, district workforce budget caps and common perceptions of nepotism and corruption in recruitment decisions. Previous studies have reported the constraints encountered in health workforce recruitments in decentralized systems in Uganda, Ghana, Tanzania and Nigeria (Ssengooba *et al.*, 2007; Munga *et al.*, 2009; Sakyi, 2013; Frumence *et al.*, 2013; Mbemba *et al.*, 2016; Sumah and Baatiema, 2019).

Our study does however highlight the potential influence of ‘change agents’ in driving health system reform and in unlocking fiscal space for health in a resource-constrained setting. At an institutional level, we found that the presence of transition ‘champions’ at multiple levels including within district governance systems but also at the facility level was a key enabler of increasing budgetary allocations for expanding the health workforce in Uganda. We find that PEPFAR support had a ‘trigger effect’ that synergized the role of internal ‘champions’ in promoting HSS. In influencing HSS in Uganda, PEPFAR can be said to have been acting as a catalyst. The role of ‘external change agents’ is recognized in implementation research (Means *et al.*, 2020). Our findings add to the accumulating evidence base pointing to the influence of leadership and governance on health systems development and outcomes in decentralized settings (Mitchell and Bossert, 2010; Abimbola *et al.*, 2019; Schneider *et al.*, 2020).

This study had a limitation that we wish to acknowledge. Due to budgetary limitations, we were unable to select an equal number of ‘high absorber’ and ‘lower absorber’ districts to enable a more ideal comparative case-study analysis. However, the common attributes across our ‘low absorber’ cases such as the absence of transition ‘champions’ and a lack of prioritization of the health workforce in the district wage bill suggests that these attributes could be common across a larger sample of ‘low absorber’ districts.

## Conclusion

Our case studies offer implementation research lessons on effective donor transition and insights into pragmatic strategies for increasing public spending on expanding the health workforce in a low-income setting.

## Supplementary Material

czab077_Supp

## Data Availability

The qualitative data underpinning this study are not publicly available due to ethical reasons but reasonable requests can be made to the first author.
