# Supplementary material for: Transitioning health workers from PEPFAR contracts to the Uganda government payroll
Source: Health Policy Plan. 2021 Jul 8;36(9):1397–407. doi: 10.1093/heapol/czab077 (PMC8505860; doi:10.1093/heapol/czab077)
Supplement: czab077_Supp [file czab077_supp.zip › HPPms_Figure 1.docx]

**Figure 1: Number of Health workers absorbed in ‘high absorber’ districts.**
