# Supplementary material for: Transitioning health workers from PEPFAR contracts to the Uganda government payroll
Source: Health Policy Plan. 2021 Jul 8;36(9):1397–407. doi: 10.1093/heapol/czab077 (PMC8505860; doi:10.1093/heapol/czab077)
Supplement: czab077_Supp [file czab077_supp.zip › HPPms_Figure 2.docx]

**Figure 2: Number of Health workers transitioned between 2014 and 2017**
