# Supplementary material for: Transitioning health workers from PEPFAR contracts to the Uganda government payroll
Source: Health Policy Plan. 2021 Jul 8;36(9):1397–407. doi: 10.1093/heapol/czab077 (PMC8505860; doi:10.1093/heapol/czab077)
Supplement: czab077_Supp [file czab077_supp.zip › HPPms_Table 2.docx]

| **Table 2:** Category of participants | | | |
| --- | --- | --- | --- |
| **Respondent type** | **Round 1** | **Round 2** | **Total** |
| High-level sector ministry technocrats | 14 | 0 | 14 |
| District Health Team leaders | 12 | 3 | 15 |
| Facility in-charges/ managers | 18 | 4 | 22 |
| Representatives of regional-based PEPFAR Implementing Partners (IPs) | 11 | 2 | 13 |
| U.S. embassy program officers (USAID and CDC) | 3 | 0 | 3 |
| **Focus Group Discussions** | 6 | 2 | 15 |
| Transitioned health workers | 75 | 12 | 87 |
