# Supplementary material for: Transitioning health workers from PEPFAR contracts to the Uganda government payroll
Source: Health Policy Plan. 2021 Jul 8;36(9):1397–407. doi: 10.1093/heapol/czab077 (PMC8505860; doi:10.1093/heapol/czab077)
Supplement: czab077_Supp [file czab077_supp.zip › HPPms_Table 4.docx]

**Table 4: Cadres of health workers transitioned from PEPFAR to Government of Uganda**

| **HEALTH WORKER CADRE** | **No. of transitioned HWs** | **% by HW cadre N=694** |
| --- | --- | --- |
| Enrolled Nurse | 275 | 39.6 |
| Enrolled Midwife | 204 | 29.4 |
| Medical Laboratory Technician | 54 | 7.8 |
| Medical Clinical Officer | 50 | 7.2 |
| Biostatistician | 35 | 5.0 |
| Medical Officer | 30 | 4.3 |
| Nursing Officer Nurse | 14 | 2.0 |
| Medical Laboratory Technologist | 13 | 1.9 |
| Enrolled comprehensive Nurse | 8 | 1.2 |
| Nursing Officer Midwife | 6 | 0.9 |
| Dispenser | 3 | 0.4 |
| Pharmacist | 1 | 0.1 |
| Medical Records Assistant | 1 | 0.1 |
| Laboratory Assistant | 0 | 0.0 |
| Anesthetic Officer | 0 | 0.0 |
| **Total** | **694** | **99.9** |
