# Supplementary material for: Transitioning health workers from PEPFAR contracts to the Uganda government payroll
Source: Health Policy Plan. 2021 Jul 8;36(9):1397–407. doi: 10.1093/heapol/czab077 (PMC8505860; doi:10.1093/heapol/czab077)
Supplement: czab077_Supp [file czab077_supp.zip › HPPms_Table 1.docx]

**Table 1: Characteristics of Case-study Districts**

| **Geographic Sub-region of Uganda** | **District** | **HIV Prevalence (%)[**[**12**](#_ENREF_12)**]** | **Inclusion Criteria** |
| --- | --- | --- | --- |
| South Western | Sheema | 7.7 | High number of transitioned HWs & high HIV prevalence |
| East Central | Iganga | 4.4 | High number of transitioned HWs, mixed rural -urban |
| Mid- East | Tororo | 4.8 | High number of transitioned HWs & cross border dynamics, mainly urban |
| Mid -West | Kasese | 5.5 | High number of transitioned HWs & cross border dynamics, mainly urban |
| Central 2 | Mubende | 7.4 | High number of transitioned HWs & high HIV prevalence |
| Mid North | Nwoya and  Apac | 7.0 | High number of transitioned HWs & high HIV prevalence, largely rural |
| North East | Napac | 3.4 | High number of transitioned HWs & hard to reach, rural |
| Kampala | Kampala | 6.6 | Capital city& houses many agencies involved in transition planning |
