# Supplementary material for: Transitioning health workers from PEPFAR contracts to the Uganda government payroll
Source: Health Policy Plan. 2021 Jul 8;36(9):1397–407. doi: 10.1093/heapol/czab077 (PMC8505860; doi:10.1093/heapol/czab077)
Supplement: czab077_Supp [file czab077_supp.zip › HPPms_Table 3.docx]

**Table 3: Milestones in the HW transition implementation process**

| **Level & Stakeholders** | **Key actions** |
| --- | --- |
| National | - MoU between PEPFAR and GoU - Harmonization of salaries - Inter-sector transition meetings around a road map Developing a transition road map |
| District | - Joint Planning by regionally-based IPs & District actors. - Determining district HRH needs - Wage bill analysis - Health worker recruitment and deployment |
| Health facility level | - Health worker orientation - HW performance management during the contract phase |
